# The attitude-behaviour gap in biosecurity: Applying social theories to understand the relationships between commercial chicken farmers' attitudes and behaviours

**DOI:** 10.3389/fvets.2023.1070482

**Published:** 2023-02-09

**Authors:** Hai-ni Pao, Elizabeth Jackson, Tsang-sung Yang, Jyan-syung Tsai, Yi-ting Hwang, Watson H. T. Sung, Dirk U. Pfeiffer

**Affiliations:** ^1^Veterinary Epidemiology, Economics and Public Health Group, Department of Pathobiology and Population Sciences, Royal Veterinary College, Hatfield, United Kingdom; ^2^School of Management and Marketing, Curtin University, Perth, WA, Australia; ^3^Independent Researcher, New Taipei City, Taiwan; ^4^Department of Finance and Cooperative Management, National Taipei University, New Taipei City, Taiwan; ^5^Department of Statistics, National Taipei University, New Taipei City, Taiwan; ^6^Agricultural Bank of Taiwan, New Taipei City, Taiwan; ^7^Centre for Applied One Health Research and Policy Advice, Jockey Club College of Veterinary Medicine and Life Sciences, City University, Kowloon, Hong Kong SAR, China

**Keywords:** cognitive consistency, commercial chicken farms, content analysis, mixed-methods research, biosecurity

## Abstract

**Introduction:**

Traditionally, it is believed that people's behaviours align with their attitudes; however, during COVID-19 pandemic, an attitude-behaviour gap in relation to preventive measures has been observed in recent studies. As such, the mixed-methods research was used to examine the relationships between farmers' biosecurity attitudes and behaviours in Taiwan's chicken industry based on the cognitive consistency theory.

**Methods:**

Content analysis of face-to-face interviews with 15 commercial chicken farmers identified their biosecurity responses to infectious disease threats.

**Results:**

The results indicated the mismatch of farmers' attitudes and behaviours towards specific biosecurity measures, in that they act differently than they think. The findings of the qualitative research allowed the research team to conduct the subsequent quantitative, confirmatory assessment to investigate the mismatch of farmers' attitudes and behaviours in 303 commercial broiler farmers. Survey data were analyzed to discover the relationships between farmers' attitudes and behaviours in relation to 29 biosecurity measures. The results show a mixed picture. The percentage of the farmers who had the attitude-behaviour gap towards 29 biosecurity measures ranged from 13.9 to 58.7%. Additionally, at the 5% significant level, there is an association between farmers' attitudes and behaviours for 12 biosecurity measures. In contrast, a significant association does not exist for the other 17 biosecurity measures. Specifically, out of the 17 biosecurity measures, the disconnection of farmers' attitudes and behaviours was observed in three specific biosecurity measures such as using a carcass storage area.

**Discussion:**

Based on a fairly large sample of farmers in Taiwan, this study confirms the existence of an attitude-behaviour gap in context and applies social theories to provide an in-depth understanding of how infectious diseases are managed in the animal health context. As the results demonstrate the necessity of tailoring biosecurity strategies to address the gap, it is time to reconsider the current approach by understanding farmers' real attitudes and behaviours in relation to biosecurity for the success of animal disease prevention and control at the farm level.

## 1. Introduction

Epidemic animal diseases threaten livestock productivity and the wider society (1). Zoonotic diseases that threaten human health (2) by direct or indirect contact with animals have become of great concern to the global community (3). Six in ten infectious human diseases are transmitted from animals (4), and three in four emerging infectious diseases are zoonotic (4, 5). National authorities have faced the challenge of developing integrated strategies for effective prevention and control of animal diseases (6, 7). Siekkinen et al. (8) emphasised that on-farm biosecurity is a critical component of disease prevention and control. Recently, studies have applied both quantitative and qualitative approaches to understand farmers' knowledge, attitudes, and underlying drivers related to on-farm biosecurity practises (9–17). Studies also discovered how social cues, messages of infection risk and message delivery methods affect farmers' compliance with biosecurity (18–21).

**Figure 1:**
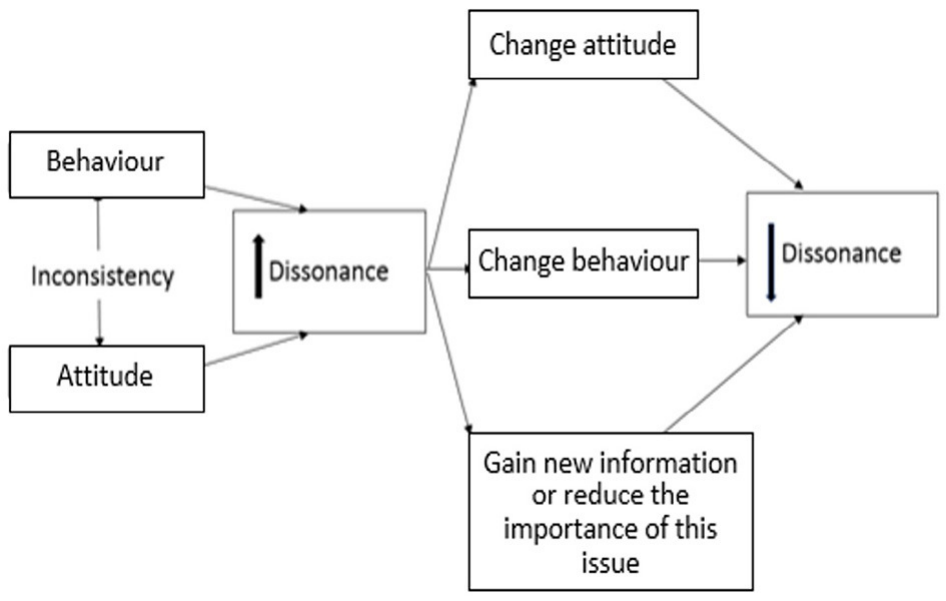
The underlying mechanisms of cognitive dissonance. When an individual's behaviour is inconsistent with his attitude, the tension of cognitive dissonance will increase unless the individual changes his/her attitude or behaviour. Alternatively, the individual may try to gain new information or reduce the importance of this issue.

In the agricultural literature, an increasing number of studies have applied behavioural change theories to explain farmers' decision-making process in relation to on-farm biosecurity (17, 22–24). The cognitive consistency theories have been applied to understand the logic behind people's decision-making process and the theories describe how meanings and judgments influence an individual's feelings, actions, and adoption (25). However, gaps in current knowledge persist regarding the relationships between farmers' attitudes and behaviour towards biosecurity. Farmers' attitudes have been shown to affect farm management performance (26). Farmers with awareness and concerns about water quality have better practises in water management. Casal et al. (27) also stated that farmers' perception of a given biosecurity measure is associated with their current practise on the farm. Attitudes may have higher impacts on relevant behaviours when farmers' attitudes are consistent with their beliefs, which are constructed from high amounts of relevant information and personal experience (28). Enticott (29) also suggested that farmers' attitudes have a statistically significant positive association with their behaviours. In contrast, with the example of peach growers, Mankad (22) explained the consequence of cognitive dissonance. Amongst all existing cognitive consistency theories, the cognitive dissonance theory (30) bears great significance in the effort to better understand people's attitudes to their behaviours. Figure 1 shows the underlying mechanisms of cognitive dissonance. When receiving new information about the invasion risk of Queensland fruit fly from farmer group advisers, if this new information leads to conflicts with farmers' current beliefs, it is likely that farmers will respond negatively by not attending forums in relation to this issue, ignoring this new information or considering that proactive biosecurity practises are not necessary. The outcome, of course, is a heightened invasion risk.

Due to the pandemic of COVID-19, a considerable amount of research has shown that a variety of factors influence people's intentions in practising COVID-19 preventive behaviours (31–35). There is also increasing debate over the importance of a socio-cultural approach to better understand infectious diseases (36). In addition, an attitude-behaviour gap in relation to COVID-19 preventive measures has been observed in recent studies (37–39). Fischer et al. (37) also observed that, when governments enforce their people to adhere to certain COVID-19 preventive behaviours, cognitive dissonance will be induced. We suggested that this is very much the case when it comes to biosecurity policy or relevant management recommendations at farm level. As Pao et al. (17) suggested that the relationships between attitudes and behaviours amongst farmers are complex, conflicts between farmers' biosecurity attitudes and behaviours will lead to their physical and psychological tension when conducting biosecurity practises.

Taiwan is located in East Asia and its neighbouring countries include China, Japan, Korea, Vietnam and the Philippines etc. There are around 100 million chickens and 6,200 chicken farms in Taiwan. The fact that Taiwan has limited farmland has resulted in intensive poultry rearing systems (land use for poultry is 1,199 ha). To mitigate the risk of potential infectious disease transmission and outbreaks within the national poultry flock, Taiwan's government has established biosecurity procedures for poultry disease management at the farm level for two decades. Taiwan's chicken farmers have received the Taiwan government's biosecurity training education for more than a decade. However, despite this, there have been outbreaks caused by avian influenza H5 strains in Taiwan over the past decade (40). Pao et al. (17) discovered that the decision of Taiwan's commercial chicken farmers to adopt biosecurity measures was influenced by a variety of internal and external factors. As farmers have a diverse range of needs and restrictions when it comes to their farming enterprises, it is difficult to ensure farmers continually recognise and implement biosecurity measures that will protect their own and livestock's health. As such, this study hypothesised that a gap in farmers' biosecurity attitudes and behaviours may exist and put farmers' and livestock's health at risk. For example, if farmers took desirable biosecurity actions despite not having a positive attitude towards the importance of biosecurity, they might change their behaviours if there is a lack of resources. In this study, the cognitive consistency theory was used to examine the associations between farmers' attitudes and behaviours towards biosecurity, and relevant social theories were applied to explain the relationships between farmers' attitudes and behaviours.

## 2. Materials and methods

**Figure 2:**
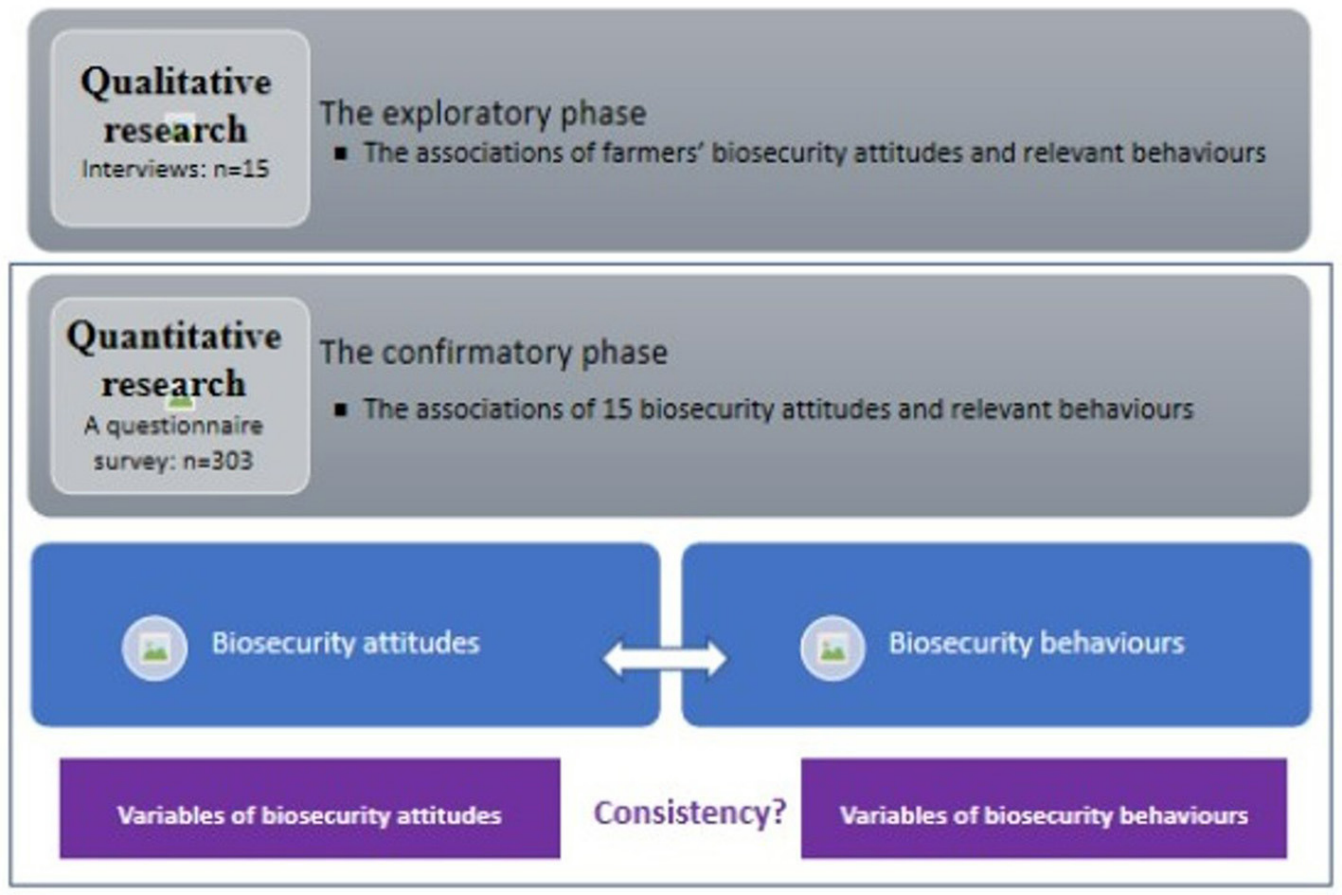
The framework and the relationships of variables used in the study. The study involved two consecutive phases: qualitative research followed by quantitative research. The qualitative phase served as exploratory research so that the relationships of farmers' biosecurity attitudes and behaviours could be examined to inform the subsequent quantitative research.

The framework and the relationships of variables used in the study are presented in Figure 2. The study involved two consecutive phases: qualitative research followed by quantitative research. As this study aimed to examine if an attitude-behaviour gap might exist in relation to biosecurity at the farm level, the cognitive consistency theory was used as the theoretical basis of the research. Based on the theory, the hypothesis was proposed:

- Null hypothesis (*H*0): farmers' attitudes and behaviours in relation to a specific biosecurity measure are not demonstrated to be significantly associated.
- Alternative hypothesis (*H*a): farmers' attitudes and behaviours in relation to a specific biosecurity measure are demonstrated to be significantly associated.

The qualitative phase served as exploratory research so that the relationships of farmers' biosecurity attitudes and behaviours could be examined to inform the subsequent quantitative research; the latter being the confirmatory phase to formally assess the attitude-behaviour gap.

### 2.1. The qualitative research

This research was part of a two-tier research initiative that investigated the mindset of farmers in pursuing on-farm biosecurity (17, 41). Upon approval from the Ethics and Welfare Committee of the Royal Veterinary College, University of London, the United Kingdom (approval #URN 2014 0116H), participating farmers were recruited through local disease control centres (LDCCs), the Poultry Association, and private feed companies. Theoretical sampling was chosen to select participants based on a specific characteristic: at least 10 years of chicken farming experience.

An interview guide was prepared [(42), p. 16] with simple, short open-ended questions to encourage the participants to express their views for exploring the current situation of on-farm biosecurity practises in Taiwan. Interviews were conducted on farms for each participant's convenience. The interviews were conducted in Mandarin within one and a half to 2 h, and data were translated to English and typed into Word (Windows XP). Descriptive analyses and qualitative content analysis were performed (43–45). Open coding of respondents' responses was performed to develop major themes and subthemes. Data were double-checked and peer debriefing was repeated to refine the themes and improve the validity and reliability of the data (44, 46, 47).

### 2.2. The quantitative research

As recommended by Creswell (47) the results of the qualitative research were combined with recent knowledge collected from the literature review and opinions from Taiwan's poultry and epidemiology experts to develop a questionnaire which consisted of simple and closed questions. The questions were based on factors related to on-farm biosecurity status considered relevant in the literature, including risk factors for the introduction and transmission of poultry diseases in Taiwan. A total of 29 biosecurity measures were considered important and should be conducted continuously. The answer of “Yes” means that the specific biosecurity measure was conducted for each batch.

In addition, the section in relation to farmers' attitudes towards different biosecurity measures consisted of a five-point Likert scale. The scale ranged from 1 to 5; that is, 1 = “highly unimportant,” 2 = “unimportant,” 3 = “neutral,” 4 = “important” and 5 = “highly important.” The questionnaire provided options of “Neutral” and “No idea” to avoid the bias that respondents do not want to answer or do not understand the question (48). Their responses were further divided into two groups: (1) “Recognising the importance of biosecurity measures” that included the responses of “Highly important & Important” of the five-point Likert scale; (2) “Not recognising the importance of biosecurity measures” that included the responses of “Neutral and Unimportant and Highly unimportant” of the five-point Likert scale.

The survey was approved by the Ethics and Welfare Committee of the Royal Veterinary College, University of London, the United Kingdom (Approval # URN 2015 0125H). A pilot study involving 13 participants familiar with Taiwan broiler production systems was completed prior to the main study. The study populations included commercial broiler farmers from a variety of flock sizes and farm types but excluded backyard farmers due to very few numbers of these farmers after urbanisation for decades. Due to outbreaks of HPAI (highly pathogenic avian influenza) in waterfowl during the data collection period, the willingness of farmers to participate in the research was low; therefore, a convenience sampling method had to be adopted. This was based on the following approach: In addition to face-to-face interviews when the LDCC officials conducted on-farm inspections, the officials also distributed questionnaires to commercial broiler farmers when farmers gathered together for a meeting or training. Moreover, due to restrictions of movement during outbreaks of HPAI, responses from HPAI-infected farms and surrounding farms within a three-kilometre radius were also obtained over the phone in order to reduce sampling bias.

Commercial broiler farmers were given a detailed explanation to ensure their understanding of the purpose of the study. Participants were assured that their contribution to the study was entirely voluntary, all data would be anonymous and stored securely, and any participant was free to withdraw from the study at any time without prejudice. Prior to the survey, verbal consent from each participant was obtained, and any information which potentially could lead to their identification was removed.

Data cleaning methods (49, 50) were applied to verify structural stability such as responses and non-response were labelled accurately. Invalid entries were also identified. Respondents had to complete at least 80% of each section in the questionnaire. All analyses were performed using the statistical software package SPSS 22.0 for Windows (51). The statistical analyses were based upon two-way contingency tests (Fisher's exact test if expected frequency < 5) using the Crosstabs procedures to test the associations between farmers' biosecurity attitudes and their behaviours. For specific biosecurity measures that farmers' attitudes and behaviours could not be demonstrated to be significantly associated, McNemar's test was further used to test the consistency in responses across two variables which can examine whether attitudes variables had effect on behaviour variables.

## 3. Results

As the phenomena of the mismatch of farmers' attitudes and behaviours in relation to biosecurity measures were discovered in the qualitative research, the questionnaire survey (*n* = 303) reconfirmed the mismatch of farmers' attitudes and behaviours towards biosecurity in a larger population sample.

### 3.1. The qualitative research

Fifteen recommended potential participants who had at least 10 years of chicken farming experience were recruited by telephone to ensure their understanding of the purpose and the procedure of the study, including data anonymisation and security, and seven of them declined to be interviewed. Alternatively, seven farmers with at least 20 years of chicken farming experience were recruited. As the main form of chicken farming in Taiwan is family production with men being the primary decision-makers (52), only male farmers (*n* = 15) joined this study and they all reaffirmed that they were their farm's leading decision-maker. Supplementary Table 1 provides an overview of the characteristics of the farmers interviewed. Almost all farm owners (14/15) have developed their biosecurity strategies to decrease the risks of disease outbreaks. Theoretical saturation of the data was reached after 15 interviews where the additional interview did not yield any new knowledge to address the research questions (53–55). Themes and subthemes emerging from the interview data are summarised in Table 1.

**Table 1:** Summary of themes emerging from the interview data (*n* = 15).

**Major themes (The phenomenon observed)**	***n***	**Subthemes**	***n***
A positive attitude towards biosecurity together with desirable biosecurity actions	13	1. Having done a lot and trusting in those biosecurity measures 2. Vaccine programme	8
			9
Neither having a positive attitude towards biosecurity nor taking desirable biosecurity actions	3	1.A loading area 2. Vehicles shipping broilers for sale always empty upon arrival /Transport the chickens by themselves/ Shipping cages and collection buckets always empty upon arrival at the farm 3. A transition zone 4. Fixed supply of chicks	1
			1
			1
			1
A positive attitude towards biosecurity without taking desirable biosecurity actions	5	1. Downtime control 2. Cleaning and disinfection of vehicles upon arrival at the farm 3. A contracted veterinarian	3 1 2
Without a positive attitude towards biosecurity but taking desirable biosecurity actions	4	1. Anti-bird netting 2. Cleaning and disinfection of shipping cages and buckets upon arrival at the farm	3
			2

Five farmers thought the effectiveness of biosecurity is determined by their attitudes and precaution measures as “*The key to the success of biosecurity is farmers” (White-chicken broiler farmer, Interview 1)*. However, due to Taiwan's high-density farm systems and the circulated variants of avian influenza, two farmers perceived the risks of avian influenza as uncontrollable as “*Bird flu will happen definitely because the government cannot control it.” (Indigenous chicken farmer, Interview 6) and “From other farmers' experiences, I believe that it is hard to prevent bird flu.” (Indigenous chicken farmer, Interview 5)*.

Thirteen farmers expressed that they were willing to implement a certain amount of biosecurity measures, and they were confident in their current biosecurity practises as “*I've done a lot [biosecurity measures]; thus, I do not worry about avian influenza disease.” (White-chicken broiler farmer, Interview 6)*. They also expressed their appreciation about what they had learned in biosecurity:

‘*The more [biosecurity] courses they provide, the better I can learn. The private feed companies and the Poultry Associations will offer free training courses in relation to biosecurity. [...] Veterinary schools will also notify us to attend their courses.' (Indigenous chicken farmer, Interview 1)*

‘*Young or educated farmers will attend seminars or discuss with others to improve their knowledge about biosecurity. [...] Most white chicken farmers have done their best in biosecurity, and we have the fewest avian influenza cases.' (White-chicken broiler farmer, Interview 1)*

Conversely, three farmers were not willing to do specific biosecurity measures due to the impracticality and ineffectiveness of the measures as “*Experts suggest that it is crucial to set up a transition zone at the farm for preventing infectious diseases, but it is impossible for us to set up it. We just don't have extra space. We also don't believe it works for avian influenza.” (Indigenous chicken farmer, Interview 4)*. They also expressed their intention to ignore the perceived risks of infectious diseases, particularly for avian influenza:

‘*The government suggests that we should transport the chickens by ourselves; otherwise, we should ensure that, when trucks [shipping broilers for sale] enter our farms, the trucks or all shipping cages are empty, but this is impossible. We cannot transport the chickens by ourselves, and we don't know whether the trucks or their cages are empty or not when they arrive. In addition, I don't think it is necessary because after the trucks take the chickens away, our farms are empty.' (Indigenous chicken farmer, Interview 2)'*

Additionally, the phenomena of the mismatch of farmers' attitudes and behaviours in relation to biosecurity were observed in two ways:

(1) Four farmers complained about the practicalities of current biosecurity regulations although they still followed those regulations such as cleaning and disinfection of shipping cages and buckets upon arrival at the farm. In particular, farmers all knew that a farm without evaporative cooling systems shall establish anti-bird netting to prevent transmission of avian influenza from wild bird contact which was governed by the laws; however, disagree with the necessity of anti-bird netting as a biosecurity strategy due to several reasons such as the effectiveness of the netting in preventing bird contact as “*Poor outcomes for anti-bird netting.” (White-chicken broiler farmer, Interview 1):*

‘*The government enforces us to install anti-bird netting, but we have problems. Some birds come in, but they do not have ways to go out.' (Indigenous chicken farmer, Interview 4)*

‘*The government has announced that, if there is no anti-bird netting, the farmer cannot get any compensation for their chickens culled (because of avian influenza). But many farmers think the anti-bird netting is useless to prevent avian influenza.' (White-chicken broiler farmer, Interview 1)*

(2) Five farmers admitted that because of potential restrictions such as time and money, some biosecurity measures have not been carried out despite the recognised importance of those measures. For example, intensive livestock producers are legally required to employ a contracted veterinarian to help manage the risk of disease outbreaks. Although all participants knew that the requirement of a contracted veterinarian or an employed veterinarian was meant to help them, two farmers still expressed their unwillingness to enter into a contract with a veterinarian due to cost concerns *as “Having a contracted veterinarian is only for the purpose of obeying the policy. We do not have money to employ veterinarians. [...] We seek for the veterinarian's assistance only when we need a final diagnosis.” (White-chicken broiler farmer, Interview 1)*. In addition, trucks delivering feed and chicks are the most concerning issues related to transmitting disease upon farmers' expression, but they don't have enough resources to conduct this biosecurity measure:

‘*We know that trucks such as those delivering feed will transmit diseases, but we cannot do disinfection each time. We don't have enough time and labour to do this*.' *(Indigenous chicken farmer, Interview 3)*

### 3.2. The quantitative research

Seven LDCCs in different cities/counties of Taiwan were willing to participate in the study, and a total of 335 farmers responded to the survey. The seven cities/counties covered 61.87% (2,440/3,944) of all commercial broiler farms in Taiwan. Amongst the 2,440 commercial broiler farm owners, 13.73% of the owners (335/2,440) were recruited for the study.

After removing invalid responses from the data set, 303 responses were retained for further analyses. Most broiler farmers participating in the study were more than 30 years old, and over 75% of the farmers had more than 10 years farming experience. Supplementary Table 2 shows the general characteristics of farms and farmers. The mean chicken number per farm was 29,663 ± 1,419 and the mean batch number of the farms (the number of flocks produced per year) was 3.9 ± 0.1. About 80% of the farmers raised their chickens indoors and about 65% of them have senior high school degrees (or higher).

Table 2, Supplementary Tables 3, 4 provide the abbreviates and descriptive statistics of farmers' attitudes and behaviours in relation to biosecurity. Most farmers recognised the importance of the 15 biosecurity measures. Obvious variations in the adoption of specific biosecurity measures were observed; that is, while 96.7% of the farmers implemented the measure of “B21 ManureMoved,” only 5.3% of the participant conducted the measure of “B29 FeedQuality.”

**Table 2:** Descriptive statistics of farmers' attitudes and behaviours in relation to biosecurity (*n* = 303).

**Code**	**Variable**	**Abbreviation**	**Category**
**(A) Farmers' attitudes**			
A1	The implementation of vaccination programmes	PercVaccineProgramme	1. Recognising the importance of biosecurity measures (including the responses of “Highly important and Important” of the five-point Likert scale) 2. Not recognising the importance of biosecurity measures (including the responses of “Neutral& Unimportant &Highly unimportant” of the five-point Likert scale)
A2	The disinfection of chicken houses	PercDisinfectedChickHouse	
A3	Removal for manure and dead animals	PercRemovedManure	
A4	The disinfection of personnel and vehicles	PercDisinfectedPersonnel&Vehicle	
A5	The management of diseased chickens	PercDiseasedChick	
A6	Entrance control of personnel and vehicles	PercEnteranceControl	
A7	All-in-all-out measures	PercAllInAllOut	
A8	The disinfection of equipment	PercDisinfectedEquipment	
A9	The disinfection of shipping cages and buckets	PercDisinfectedCage	
A10	Vermin and wild bird control	PercVermitControl	
A11	Movement control of chickens between chicken houses	PercChickControl	
A12	Ensured water and feed quality	PercWater&FeedQuality	
A13	A barrier between clean area and dirty area in the farm	PercTransitionZone	
A14	Fixed suppliers of chicks	PercChickFixedSupply	
A15	A separate loading area in the farm	PercLoadArea	
**(B) Farmers' behaviours**			
B1	Poultry house completely disinfected after each production round	DisinfectedChickHouse	Yes^†^/No
B2	A sanitary transition period after each production round	SanitaryPeriod	Yes^†^/No
B3	Vaccination programmes implemented	VaccineProgramme	Yes^†^/No
B4	“All-in- all-out” management strictly implemented in the farm	AllInAllOut	Yes^†^/No
B5	Disinfectant, gloves, shoe covers or taking baths “strictly” used for the cleaning and disinfection of personnel before entering animal housing	DisinfectedPersonnelEnterance	Yes^†^/No
B6	Cleaning and disinfection of vehicles upon arrival at the farm	DisinfectedVehicle	Yes^†^/No
B7	Anti-bird netting placed for the poultry houses	BirdNetting	Yes^†^/No
B8	Disinfectant, gloves, shoe covers or taking baths “strictly” used for the cleaning and disinfection of personnel upon arrival	DisinfectedPersonnel	Yes^†^/No
B9	Vehicles shipping broilers for sale always empty upon arrival at the farm	VehicleArrivalBroilerEmpty	Yes^†^/No
B10	Downtime control implemented while personnel arrive at the farm	DowntimeControl	Yes^†^/No
B11	Using a transition zone in the farm	TransitionZone	Yes^†^/No
B12	Broilers moved between poultry houses	ChickMovedBetweenHouse	No^†^/Yes
B13	Using a separate loading area in the farm	LoadArea	Yes^†^/No
B14	The disinfection of equipment strictly conducted while moving between different poultry houses	DisinfectedEquipmentBetweenHouse	Yes^†^/No
B15	The disinfection of equipment after use strictly conducted	DisinfectedEquipmentAfterUse	Yes^†^/No
B16	Disinfection measures taken for equipment before entering the farm	DisinfectedEquipmeentBeforeEntering	Yes^†^/No
B17	New needles or disinfect needles while vaccinating chicks between different poultry houses	DisinfectedNeedleBetweenHouse	Yes^†^/No
B18	Dead chickens taken out from poultry houses for 2 times or more than 2 times per day	FrequencyChickDisposal	Yes^†^/No
B19	Cleaning and disinfection of shipping cages and buckets upon arrival at the farm	CageDisinfectedArrivial	Yes^†^/No
B20	Diseased animals always handled after the healthy ones	DiseasdChickHandledAfterHealthyChick	Yes^†^/No
B21	Manure removed	ManureMoved	Yes^†^/No
B22	Cleaning and disinfection of shipping cages and buckets upon before entering animal housing	CageEntering	Yes^†^/No
B23	Diseased animals isolated from healthy ones	DiseasedChickIsolation	Yes^†^/No
B24	Shipping cages and collection buckets always empty upon arrival at the farm	CageEmpty	Yes^†^/No
B25	Using a carcass storage area	CarcassStored	Yes^†^/No
B26	Chicks originate from fixed suppliers	ChickFixedSupply	Yes^†^/No
B27	Using a manure storage area	ManureStored	Yes^†^/No
B28	The quality of chickens' drinking water checked by bacteriological analysis	WaterQuality	Yes^†^/No
B29	The quality of feed checked by bacteriological analysis	FeedQuality	Yes^†^/No

† The desirable action of a biosecurity behaviour.

**Figure 3:**
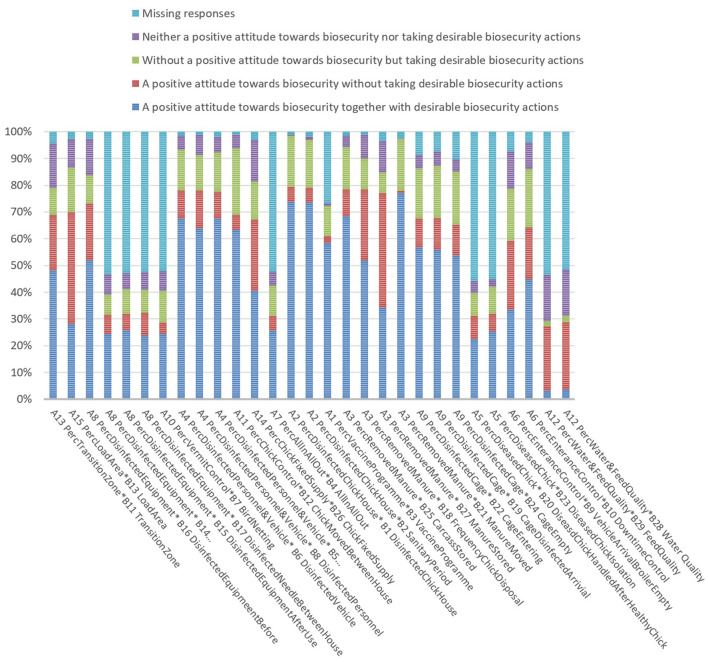
The variety of the relationships between farmers' biosecurity attitudes and relevant behaviours. While some farmers tended not to translate their knowledge into the relevant biosecurity actions despite recognising the importance of biosecurity measures, a number of farmers still took desirable biosecurity actions despite not having a positive attitude towards the importance of biosecurity.

Contingency tables described the associations between attitude variables and their relevant behaviour variables. Supplementary Table 5 provides the descriptive statistics of the associations between farmers' biosecurity attitudes and the relevant biosecurity behaviours. As shown in Figure 3, there is a variety of the relationships between farmers' biosecurity attitudes and relevant behaviours:

- A positive attitude towards biosecurity together with desirable biosecurity actions: The percentage of the farmers who conducted desirable biosecurity actions together with a positive attitude towards biosecurity ranged from 3.3 to 73.9% for the 29 biosecurity measures. The former was the variable of “B29 FeedQuality”; the latter was the variable of “B1 DisinfectedChickHouse.”
- Neither having a positive attitude towards biosecurity nor taking desirable biosecurity actions: The percentage of the farmers who had neither a positive attitude towards biosecurity nor took desirable biosecurity actions ranged from 0.3 to 17.2% for the 29 biosecurity measures. The former was the variable of “B1 DisinfectedChickHouse”; the latter was the variable of “B28 WaterQuality” and “B29 FeedQuality.”

- A positive attitude towards biosecurity without taking desirable biosecurity actions: The percentage of the farmers who tended not to translate their knowledge into relevant biosecurity actions despite recognising the importance of biosecurity measures ranged from 0.7 to 42.9% for the 29 biosecurity variables. The former was the variable of “B21 ManureMoved”; the latter was the variable of “B27 ManureStored.”
- Without a positive attitude towards biosecurity but taking desirable biosecurity actions: The percentage of the participants who still took desirable biosecurity actions despite not having a positive attitude towards the importance of biosecurity ranged from 2 to 25.1% for the 29 biosecurity variables. The former was the variable of “B29 FeedQuality”; the latter was the variable of “B12 ChickMovedBetweenHouse.”

As shown in Figure 3, the variable of “B1 DisinfectedChickHouse” had the fewest farmers who experienced the mismatch of their attitudes and behaviours (13.9%) while the variable of “B13 LoadArea” had the most farmers who experienced the mismatch of their attitudes and behaviours (58.7%). In addition, the proportion of missing values for the 29 biosecurity variables varied from 1.0 to 55.8%. Surprisingly, nine variables such as “B4 AllInAllOut” and “B7 BirdNetting” had a high proportion of missing values (more than 50%). That is, many farmers intended to not answer the nine questions in relation to their biosecurity practises. Additionally, the nine biosecurity measures with a high proportion of missing values also had the fewest farmers who appropriately implemented these biosecurity practises.

In summary, the percentage of the farmers who had the mismatch of their attitudes and behaviours towards the 29 biosecurity measures ranged from 13.9 to 58.7%. The results showed that, according to the participants' self-report, while some farmers tended not to translate their knowledge into the relevant biosecurity actions despite recognising the importance of biosecurity measures, a number of farmers still took desirable biosecurity actions despite not having a positive attitude towards the importance of biosecurity.

In addition, the statistical associations between farmers' biosecurity attitudes and their relevant biosecurity behaviours were listed in Table 3. The results of two-way contingency tests revealed that the statistical associations of biosecurity attitudes and behaviours in relation to 29 biosecurity measures can be divided into two groups:

- Group 1. Reject the null hypothesis at the 5% significant level (Farmers' attitudes and behaviours in relation to a specific biosecurity measure were demonstrated to be significantly associated): The associations of farmers' biosecurity attitudes and behaviours were observed in 12 biosecurity measures. Better biosecurity attitudes had a positive association with their regular biosecurity behaviours. The attitude variables included “A4 PercDisinfectedPersonnel&Vehicle,” “A8 PercDisinfectedEquipment,” “A10 PercVermitControl,” “A11 PercChickControl,” “A13 PercTransitionZone,” “A14 PercChickFixedSupply” and “A15 PercLoadArea.” The behaviour variables included “B5 DisinfectedPersonnelEnterance,” “B6 DisinfectedVehicle,” “B7 BirdNetting,” “B8 DisinfectedPersonnel,” “B11 TransitionZone,” “B12 ChickMovedBetweenHouse,” “B13 LoadArea,” “B14 EnteringDisinfectedEquipmentBetweenHouse,” “B15 DisinfectedEquipmentAfterUse,” “B16 DisinfectedEquipmeentBefore,” “B17 DisinfectedNeedleBetweenHouse,” and “B26 ChickFixedSupply.”
- Group 2. A failure to reject the null hypothesis at the 5% significant level (Farmers' attitudes and behaviours in relation to a specific biosecurity measure were not demonstrated to be significantly associated): The associations of farmers' biosecurity attitudes and behaviours were not observed in the other 17 biosecurity measures. The attitude variables included “A1 PercVaccineProgramme,” “A2 PercDisinfectedChickHouse,” “A3 PercRemovedManure,” “A5 PercDiseasedChick,” “A6 PercEnteranceControl,” “A7 PercAllInAllOut,” “A9 PercDisinfectedCage” and “A12 PercWater&FeedQuality.” The behaviour variables included “B1 DisinfectedChickHouse,” “B2 SanitaryPeriod,” “B3 VaccineProgramme,” “B4 AllInAllOut,” “B9 VehicleArrivalBroilerEmpty,” “B10 DowntimeControl,” “B18 FrequencyChickDisposal,” “B19 CageDisinfectedArrivial,” “B20 DiseasdChickHandledAfterHealthyChick,” “B21 ManureMoved,” “B22 CageEntering,” “B23 DiseasedChickIsolation,” “B24 CageEmpty,” “B25 CarcassStored,” “B27 ManureStored,” “B28 WaterQuality,” and “B29 FeedQuality.”

**Table 3:** The statistical associations of farmers' biosecurity attitudes and their relevant biosecurity behaviours.

**Variables of biosecurity attitudes**		**Variables of biosecurity behaviours**		***p*-value of the chi-square test of independence**	***p*-value of the McNemar's test**
**Group 1. Reject the null hypothesis and accept the alternative hypothesis at the 5% significant level**					
A13	PercTransitionZone	B11	TransitionZone	0.00001	
A15	PercLoadArea	B13	LoadArea	0.001	
A8	PercDisinfectedEquipment	B16 B14 B15 B17	Disinfected EquipmeentBeforeEntering DisinfectedEquipmentBetweenHouse DisinfectedEquipmentAfterUse DisinfectedNeedleBetweenHouse	0.000023 0.001 0.013 0.042	
A10	PercVermitControl	B7	BirdNetting	0.002	
A4	PercDisinfectedPersonnel&Vehicle	B6 B8 B5	DisinfectedVehicle DisinfectedPersonnel DisinfectedPersonnelEnterance	0.019 0.002 0.002	
A11	PercChickControl	B12	ChickMovedBetweenHouse	0.031	
A14	PercChickFixedSupply	B26	ChickFixedSupply	0.046	
**Group 2. A failure to reject the null hypothesis at the 5% significant level**					
A7	PercAllInAllOut	B4	AllInAllOut	0.054	0.0251
A2	PercDisinfectedChickHouse	B1 B2	DisinfectedChickHouse SanitaryPeriod	0.216 1	0.0001 < 0.0001
A1	PercVaccineProgramme	B3	VaccineProgramme	0.333	0.0001
A3	PercRemovedManure	B25 B18 B27 B21	CarcassStored FrequencyChickDisposal ManureStored ManureMoved	0.103 0.165 0.449 0.495	0.0718 < 0.0001 < 0.0001 < 0.0001
A9	PercDisinfectedCage	B22 B19 B24	CageEntering CageDisinfectedArrivial CageEmpty	0.361 0.461 0.813	0.0153 0.0240 0.0138
A5	PercDiseasedChick	B20 B23	DiseasdChickHandledAfterHealthyChick DiseasedChickIsolation	0.572 0.989	1 0.1614
A6	PercEnteranceControl	B9 B10	VehicleArrivalBroilerEmpty DowntimeControl	0.776 0.863	< 0.0001 < 0.0001
A12	PercWater&FeedQuality	B29 B28	FeedQuality WaterQuality	0.754 0.908	< 0.0001 < 0.0001

Null hypothesis (H0): Farmers' attitudes and behaviours in relation to a specific biosecurity measure are demonstrated to be significantly associated.

Alternative hypothesis (Ha): Farmers' attitudes and behaviours in relation to a specific biosecurity measure are not demonstrated to be significantly associated.

The results of this hypothesis testing revealed that, at the 5% significant level, the null hypothesis was rejected for 12 biosecurity measures such as disinfection of personnel and vehicle; however, for the other 17 biosecurity measures such as disinfection of shipping cages, there was no sufficient evidence to demonstrate the significance. Furthermore, for the 17 biosecurity measures, McNemar's test was applied to examine whether attitudes variables had effect on behaviour variables. As shown in Table 3, the disconnection of biosecurity attitudes and behaviours was observed in three biosecurity measures. The attitude variables included “A3 PercRemovedManure” and “A5 PercDiseasedChick.” The behaviour variables included “B20 DiseasdChickHandledAfterHealthyChick,” “B23 DiseasedChickIsolation” and “B25 CarcassStored.” The results confirm the existence of a gap in farmers' attitudes and behaviours in some contexts.

## 4. Discussion

The study was aimed at exploring and describing the mismatch in relation to farmers' biosecurity attitudes and behaviours from farmers' perspectives (56). Although biosecurity measures implemented on farms are mainly based on the experience and perceptions of farmers, a web of complex factors may influence farmers' biosecurity behaviours (17). While farmers encounter multifaceted barriers to implementing on-farm biosecurity, the relationships between attitudes and behaviours amongst farmers are complex. As a phenomenological study, this study utilised an exploratory sequential mixed methods approach to provide first-hand, in-depth evidence-based knowledge (47, 57). The qualitative phase included interpretations of the phenomena of the mismatch of farmers' attitudes and behaviours as well as the identification of the issues surrounding the phenomenon from farmers' hands-on farming experience. Based on the knowledge gathered from the qualitative phase, the quantitative phase provided statistical analysis to describe the gap in farmers' biosecurity attitudes and behaviours from a larger sample of the population for additional insights. Traditionally, it is believed that farmers' behaviours align with their attitudes; however, this study confirms the existence of an attitude-behaviour gap and applies social theories to provide an in-depth understanding of how infectious diseases are managed in the animal health context.

In this study, the qualitative data was first collected and analysed, and specific phenomena were observed and further used to develop the quantitative survey to further explore our research question (58–60). The qualitative phase of the mixed-methods research allowed for a deep understanding of the lived experiences of the participants' on-farm biosecurity practises. By using the results of the qualitative phase, a survey instrument was built to more accurately measure the relationships of farmer' attitudes and behaviours in relation to biosecurity. In the quantitative research, the percentage of the farmers who had the mismatch of their attitudes and behaviours towards the 29 biosecurity measures ranged from 13.9 to 58.7%. Additionally, out of 29 biosecurity measures, there is a significant association between farmers' attitudes and behaviours in relation to 12 biosecurity measures; however, there is no significant association observed for the other 17 biosecurity measures. The statistical differences in the relationships between 15 biosecurity attitude variables and 29 behaviour variables were also observed; that is, while seven attitude variables had a statistical association with 12 behaviour variables, eight attitude variables had no association with 17 behaviour variables (Table 3). While our sample contained an insufficient amount of evidence to conclude that farmers' attitudes and behaviours are associated in relation to 17 biosecurity measures such as disinfection of shipping cages, we further identified that the disconnection of biosecurity attitudes and behaviours was observed in three specific biosecurity measures, including “B20 DiseasdChickHandledAfterHealthyChick,” “B23 DiseasedChickIsolation” and “B25 CarcassStored.” Our study demonstrates that a gap in farmers' attitudes and behaviours in relation to a given biosecurity measure may exist in some contexts.

In order to connect the findings of the qualitative research with the findings of the quantitative research, a joint display (Table 4) was used to allow sample quotes of the qualitative phase to be compared to the statistical results of the quantitative phase (61). The joint display demonstrated the linking of the two phases of the study in spite of the linking nature of the sequential design in this mixed-methods research (60, 61). The qualitative interviews highlighted the existence of the mismatch of farmers' attitudes and behaviours in relation to specific biosecurity measures, which parallels the findings of the survey that there is a gap in farmers' attitudes and behaviours in relation to biosecurity, particular for the three specific biosecurity measures. Evidence from the qualitative and quantitative phases confirms the existence of a gap in farmers' attitudes and behaviours in some contexts.

**Table 4:** Joint display comparison of the findings from the qualitative and quantitative phases.

**The qualitative phase**		**The quantitative phase**
**The phenomenon observed**	**Sample quotes**	**The descriptive results (*****n*** = **303)**
A positive attitude towards biosecurity together with desirable biosecurity actions	1. *We have done everything we can do [biosecurity]. We know biosecurity is crucial for the prevention of bird flu.' (White-chicken broiler farmer, Interview 8)* 2. “*I am not afraid of any disease because of intensive vaccination.” (White-chicken broiler farmer, Interview 5)* 3. “*Experiences are very important. I believe that my farming experience provided me with the necessary expertise to determine a chicken's health condition and to decide on relevant vaccination and medication programmes for the prevention and control of infectious diseases” (Indigenous chicken farmer, Interview 4)*. 4. “*Most farmers have personal experience with avian influenza outbreaks on their farm and we also share our experiences with other farmers. With required knowledge of avian influenza and biosecurity, I believe that we have implemented the most relevant biosecurity measures that are compatible with our current husbandry practises.” (White-chicken broiler farmer, Interview 6)*	1. The percentage of the farmers who conducted desirable biosecurity actions together with a positive attitude towards biosecurity ranged from 3.3 to 73.9% for the 29 biosecurity measures. 2. 58.4% of the respondents had a positive attitude towards “the implementation of vaccine programme” and implemented “vaccine programme” for each batch.
Neither having a positive attitude towards biosecurity nor taking desirable biosecurity actions	1. “*Experts suggest that it is crucial to set up a transition zone at farm for preventing infectious diseases, but it is impossible for us to set up it. We just don't have extra space. We also don't believe it works for avian influenza.” (Indigenous chicken farmer, Interview 4)* 2. “*We don't have a separate loading area at farm. Experts told us that even if there is a loading area, it does not guarantee that we won't have bird flu. Should I spend money to rebuild my farm if zero risk is not guaranteed?” (Indigenous chicken farmer, Interview 5)* 3. “*The government suggests that we should transport the chickens by ourselves; otherwise, we should ensure that, when trucks [shipping broilers for sale] enter our farms, the trucks or all shipping cages are empty, but this is impossible. We cannot transport the chickens by ourselves, and we don't know whether the trucks or their cages are empty or not when they arrive. In addition, I don't think it is necessary because after the trucks take the chickens away, our farms are empty.” (Indigenous chicken farmer, Interview 2)'* 4. “*We will choose the source of chicks according to the quantity and quality of chicks. It is impossible and non-sense to ask us to fix suppliers.” (Indigenous chicken farmer, Interview 4)'*	1. The percentage of the farmers who had neither a positive attitude towards biosecurity nor took desirable biosecurity actions ranged from 0.3 to 17.2% for the 29 biosecurity measures. 2. 16.5% of the respondents didn't have a positive attitude towards “using a loading area,” and they didn't use a “Loading area” for each batch. 3. 10.6% of the respondents didn't have a positive attitude towards “using a loading area,” and they didn't use a “Loading area” for each batch. 4. 13.9% of the respondents didn't have a positive attitude towards “vehicles shipping broilers for sale always empty upon arrival at the farm,” and they didn't implement this measure as well. 5. 4.6% of the respondents didn't have a positive attitude towards ‘shipping cages and collection buckets always empty upon arrival at the farm', and they didn't implement this measure as well. 6. 15.5% of the respondents didn't have a positive attitude towards “fixed suppliers of chicks,” and they didn't implement this measure as well.
A positive attitude towards biosecurity without taking desirable biosecurity actions	1. “*I've raised chickens for 40 years, but my son doesn't want to take on my business. [..] It is hardly for me to do downtime control even though I know it is important. I don't have enough time and labours. Only my wife and I manage this farm.” (Indigenous chicken farmer, Interview 7)* 2. “*We know that trucks such as those delivering feed will transmit diseases, but we cannot do disinfection each time. We don't have enough time and labour to do this.” (Indigenous chicken farmer, Interview 3)* 3. “*Having a contracted veterinarian is only for the purpose of obeying the policy. We do not have money to employ veterinarians. [...] We seek for the veterinarian's assistance only when we need a final diagnosis.” (White-chicken broiler farmer, Interview 1)*	1. The percentage of the farmers who had a positive attitude towards biosecurity but didn't take desirable biosecurity actions ranged from 0.7 to 42.9% for the 29 biosecurity measures. 2. 19.5% of the respondents had a positive attitude towards “downtime control,” but they didn't implement “downtime control” for each batch. 3. 10.6% of the respondents had a positive attitude towards “cleaning and disinfection of vehicles upon arrival at the farm”, but they didn't implement this measure for each batch.
Without a positive attitude towards biosecurity but taking desirable biosecurity actions	1. “*We have to instal anti-bird netting for compensation if we have bird flu, but we don't think that anti-bird netting can prevent bird flu.” (White-chicken broiler farmer, Interview 7)* 2. “*Due to avian influenza outbreaks, the government asks us to clean and disinfect shipping cages and buckets upon arrival at the farm. But we don't think it can prevent us from infections. We still have concerns about avian influenza.” (Indigenous chicken farmer, Interview 5)*	1. The percentage of the farmers who didn't have a positive attitude towards biosecurity but still took desirable biosecurity actions ranged from 2 to 25.1% for the 29 biosecurity measures. 2. 11.9% of the respondents didn't have a positive attitude towards “anti-bird netting”, but they installed “anti-bird netting” for each batch. 3. 19.5% of the respondents didn't have a positive attitude towards “cleaning and disinfection of shipping cages and buckets upon arrival at the farm,” but they implemented this measure for each batch.

The attitude change theory can be used to explain the findings of this study. Firstly, the mismatch of farmers' attitudes and behaviours for anti-bird netting was observed in both the qualitative and quantitative phases. This phenomenon might result from “Forced compliance behaviour” (62) since this publicly forced biosecurity practises was used by the government for attitude change (63, 64). Consequently, although those farmers were enforced to instal anti-bird netting, they didn't recognise the importance of this measure. Secondly, taking the “*Indigenous chicken farmer, Interview 5”* as an example, the “Effort justification paradigm” (65, 66) and “Belief disconfirmation paradigm” (67, 68) can provide further explanations for those farmers who neither had a positive attitude towards biosecurity nor took desirable biosecurity actions. Farmers need to balance the costs of biosecurity with the expected benefits such as reducing the risks of infectious diseases (69). As such, this farmer who perceived the risks of avian influenza as uncontrollable did not have the willingness to spend money for setting up a loading zone. Since zero risk of avian influenza could not be guaranteed, he believed that the strength of a loading area is not enough to prevent avian influenza. While biosecurity information provided by the government is contrary to their beliefs, this farmer may tend to adhere to his beliefs.

On the other hand, the social exchange theory can also explain the phenomenon of mismatch between farmers' biosecurity attitudes and behaviours. The social exchange theory suggests that social behaviour is the result of an exchange process to maximise benefits and minimise costs. Taking downtime control as an example, since those farmers were not willing to pay the cost (when perceived benefits were less than potential costs or could not afford the costs, especially when lacking resources), they tended not to translate their knowledge into actions even though they recognised the importance of biosecurity practises. As a result, the exchange of biosecurity behaviours could not be achieved. There are similar findings criticised by Brennan and Christley (70) that only a small number of farmers have adopted biosecurity practises that are considered to be cost-effective and time-efficient. Additionally, Kristensen and Jakobsen (71) and Brennan and Christley (70) stated that while farmers implement biosecurity practises that are considered useless, other practises that are considered useful may be not carried out. Starting from this study, future studies could explore how direct and indirect factors influence the relationships between farmers' attitude-behaviour.

An increasing number of studies have emphasised the importance of incorporating human biosecurity behaviours and decisions to understand the link between farmers' risk attitudes, decision-making process, compliance with biosecurity and the spread of diseases (12, 72–74). As farmers' biosecurity attitudes and behaviours are related to many factors such as their social-economic background and their experience and knowledge in relation to infectious diseases (17), tailoring biosecurity strategies for farmers is essential for managing the conflicts of farmers' biosecurity attitudes and behaviours. The findings of this study suggest a lack of effective policy implementation as a result of the government placing the most effort into providing knowledge and enforcement of the policy. Inappropriate application of behavioural change theory will not result in the desired biosecurity behaviours amongst many farmers and may instead induce the mismatch of their attitudes and behaviours. It is time to reconsider the current approach by understanding farmers' real attitudes and behaviours in relation to biosecurity for the success of animal disease prevention and control at the farm level.

This research did not intend to identify any series of feedback loops from any given biosecurity behaviour to the attitudes. Instead, this research examined the associations of farmers' biosecurity attitudes and behaviours with the cognitive consistency theory. Our findings suggest that continuous efforts to ensure farmers' implementation need to be established through a full understanding of and timely response to farmers' needs. The findings of this study are likely to be helpful in shaping future biosecurity strategies for Taiwan's broiler farmers. Further study is required to tailor biosecurity intervention programs for managing the conflicts of farmers' biosecurity attitudes and behaviours for the success of animal disease prevention and control at the farm level. For example, the approach of targeting strategy in business may be applied to manage the differences in farmers' attitudes and behaviours.

Larsen et al. (75) suggested that there is a need in improving messaging on biosecurity and risks due to the increase in unsafe practises despite the increase in messaging about infection risks and good biosecurity while Damiaans et al. (76) and Higgins et al. (77) suggested that farmers ignore practising on-farm biosecurity due to a lack of biosecurity information. However, this is likely not the case in this study. Information channels were adequate which was recognised by the participating farmers, since authorities in Taiwan have provided biosecurity training education to the industry since 1998 (78). In addition, similar results can be found in the study of Pao et al. (17) as broiler farmers' biosecurity information is assumed to be sufficient. As such, attitude-relevant knowledge was not included in the evaluation of this study. On the other hand, in veterinary epidemiology, the effects of knowledge on attitudes towards on-farm biosecurity are also ambiguous. Ellis-Iversen et al. (79) suggested that knowledge is a factor affecting the performance of zoonotic disease control programmes in UK cattle farms; however, Delabbio et al. (80) and Palmer et al. (81) argued that biosecurity knowledge did not affect farmers' biosecurity practises in the US, Canada, and Australia. Studies also revealed that the distribution of educational documents did not enhance farmers' response to disease outbreaks, especially those provided by the governments in the UK, and Australia (9, 81). Additionally, Fabrigar et al. (82) showed that amount of knowledge did not have a significant impact on the attitude–behaviour consistency in some conditions. Stanhope and Lancaster (83) also stated that the knowledge-attitude-behaviour model ignores all the external factors constraining and influencing those choices. However, when there are concerns about participants' biosecurity knowledge, especially those in underdeveloped countries, the evaluation of farmers' biosecurity relevant knowledge still needs to be considered. The assessment of knowledge can be challenging due to unobservable complex constructs of metacognition, which could be affected by participants' verbal ability and memory capacity (84) although attitude-relevant knowledge can be evaluated by asking people to provide their subjective understandings of research objects (85). Furthermore, considering biosecurity involves a set of measures and relevant attitudes and behaviours, there might be potential restrictions to the length of survey time participants could spend for, despite the limitations of participants' verbal ability and memory capacity.

The samples for the qualitative and the quantitative phase were limited in size. While there is likely to be selection bias in that the sample overrepresented individuals being a farmer for at least 20 years in the qualitative research, the disconnection of farmers' attitudes and behaviours in relation to the three biosecurity measures exist in our fairly large sample (*N* = 303) accounting for 12.4% of the population (2,440 commercial broiler farmers). Selection bias is likely to occur in the situation that the sample overrepresented individuals with a higher level of chicken farming standards in the quantitative research. In addition, people tend to adhere to social norms even when the behaviours are inconsistent with their real attitudes (30, 86). There is a possibility that some farmers tended to report desirable biosecurity-related attitudes and actions because this study did not investigate those variables onsite. However, despite the likely presence of social desirability response bias, whereby respondents tend to report better attitudes and better biosecurity status than they practise (87), this bias is likely to be reduced by the local officials' familiarity and long-term partnerships with the research participants (88). Furthermore, a group of farmers who intended not to recognise the importance of biosecurity was observed in the qualitative and the quantitative phases, and another group of farmers who had a low implementation level of biosecurity was observed in the quantitative phase, suggesting that the social desirability response bias may only have had a minor impact on this research. As such, the sample is likely to be representative of the target population. However, further research to explore unexamined latent variables such as economic variables, the exact meaning of the missing value and the perspectives of farmers with less farming experience is useful to understand their impacts on farmers' attitudes and behaviours in relation to biosecurity. It is also crucial to further explore the underly mechanisms of the gap in farmers' attitudes and behaviours to understand their biosecurity decision-making process.

As a phenomenological study, it is unlikely to generalise the findings of this study to other areas until the results of repeated studies from other populations are similar. A replication of this study for other species or in other countries could help understand the mechanisms of farmers' on-farm biosecurity decision-making processes and allow the development of more reliable strategies for the improvement of on-farm biosecurity. The findings of this study can be applied to other developing countries in regions with similar production conditions, where biosecurity information has been delivered to farmers for many years, but there is still a need to improve the practise of biosecurity at the farm level (2). For example, similar to Taiwan, most researchers still suggest that biosecurity knowledge needs to be delivered to farmers in China (89–91). It will be more likely to apply to China's commercial broiler farmers due to the sharing of the same language and culture between Taiwan and China.

Overall, this study provides an in-depth understanding of the gap in farmers' biosecurity attitudes and their behaviours based on a fairly large sample of farm owners. The qualitative phase explored the phenomena of the mismatch of farmers' biosecurity attitudes and behaviours while the subsequent quantitative research confirmed the gap in farmers' biosecurity attitudes and behaviours in relation to biosecurity in a sample of 303 farmers. The combination of the results of the qualitative and quantitative phases provides detailed insights of the gap in farmers' attitudes and behaviours in relation to biosecurity. By integrating social theory and knowledge generated by the qualitative research with the findings of the subsequent quantitative research, this study applies social theories to explain how infectious diseases are managed in the animal health context. The findings of this study imply that the gap in farmers' biosecurity attitudes and behaviours in Taiwan will put farmers' and livestock's health at risk. This would appear to be particularly important in the context of animal health as farmers are the frontier of on-farm disease management. Traditionally, it is believed that farmers' behaviours align with their attitudes. As such, these findings suggest a lack of effective policy implementation as a result of the government placing most effort on providing knowledge and enforcement of policy. Inappropriate application of behavioural change theory will not result in the desired biosecurity behaviours amongst many farmers and may instead induce the mismatch of their attitudes and behaviours. It is time to reconsider the current approach by understanding farmers' real attitudes and behaviours in relation to biosecurity for the success of animal disease prevention and control at the farm level.

## Data availability statement

The original contributions presented in the study are included in the article/Supplementary material, further inquiries can be directed to the corresponding author.

## Ethics statement

The studies involving human participants were reviewed and approved by the Ethics and Welfare Committee of the Royal Veterinary College, University of London, United Kingdom. Written informed consent for participation was not required for this study in accordance with the national legislation and the institutional requirements.

## Author contributions

H-nP did the research and writing of the article as part of her PhD's thesis. DP, EJ, and WS were H-nP's supervisors and were involved extensively throughout all phases of the research and writing of this article. T-sY provided support in collecting data and developing the critiqued aspects of data analysis. J-sT and Y-tH provided the critiqued aspects of data analysis and presentation. All authors have given approval for the article submitted for publication.
