# Supplementary material for: The attitude-behaviour gap in biosecurity: Applying social theories to understand the relationships between commercial chicken farmers' attitudes and behaviours
Source: Front Vet Sci. 2023 Feb 9;10:1070482. doi: 10.3389/fvets.2023.1070482 (PMC9947856; doi:10.3389/fvets.2023.1070482)
Supplement: Supplementary file 1 [file Data_Sheet_1.doc]

Supplementary Information

- 1. Table S.1 Overview of the characteristics of the farm owners interviewed (n=15)

| Characteristics | Farmers (n, (%)) |
| --- | --- |
| Farm management experience | 15(100%) |
| More than 20 years | 9(60%) |
| 10-19 years | 6(40%) |
| Less than 10 years | 0(0%) |
| Farm type | 15(100%) |
| White-chicken broiler farm | 8 (53.3%) |
| Indigenous chicken farm | 7 (46.7%) |
| Farm size | 15(100%) |
| >50 thousand  Between 20 and 50 thousand  < 20 thousand | 9(60%)  6(40%)  0(0%) |
| Farm location  Northern  Central  Southern | 15(100%)  6(40%)  4(26.6%)  5(33.4%) |

Table S.2 General characteristics of the farms and farm owners in the survey (n = 303)

| Characteristics | Categories | Frequency | Effective Percentage (%) |
| --- | --- | --- | --- |
| Farm location |  |  |  |
|  | North | 48 | 15.8 |
|  | Central | 106 | 35.0 |
|  | South | 149 | 49.2 |
| Chicken type | |  |  |
|  | White-chicken broiler | 156 | 52.0 |
|  | Indigenous chicken | 144 | 48.0 |
|  | Missing | 3 |  |
| Chicken number | |  |  |
|  | <3,000 | 9 | 3.0 |
|  | 3,000-19,999 | 107 | 35.8 |
|  | 20,000-49,999 | 117 | 39.1 |
|  | > 50,000 | 66 | 22.1 |
|  | Missing | 4 |  |
| Batch number per year | |  |  |
|  | less than two batches | 17 | 5.8 |
|  | 2=<X <4 | 138 | 47.3 |
|  | 4<=X <6.5 | 130 | 44.5 |
|  | X>6.5 | 7 | 2.4 |
|  | Missing | 11 |  |
| Poultry house types | |  |  |
|  | Evaporative cooling | 116 | 38.8 |
|  | Open | 26 | 8.7 |
|  | Bird proof and kept indoor | 125 | 41.8 |
|  | Bird proof and sometimes kept outdoor | 32 | 10.7 |
|  | Missing | 4 |  |
| Farmer's highest education | |  |  |
|  | Elementary school | 31 | 10.8 |
|  | Junior high school | 70 | 24.4 |
|  | Senior high school | 118 | 41.1 |
|  | College or university | 59 | 20.6 |
|  | Postgraduate | 9 | 3.1 |
|  | Missing | 16 |  |

# Table S.3 Descriptive statistics of farmers’ attitudes towards biosecurity (n = 303)

|  | Abbreviation | Not recognising the importance of biosecurity measures†  n(%) | Recognising the importance of biosecurity measures‡  n(%) | Missing  n(%) |
| --- | --- | --- | --- | --- |
| A1 | PercVaccineProgramme | 80  (26.4%) | 217  (71.6%) | 6  (2.0%) |
| A2 | PercDisinfectedChickHouse | 59  (19.5%) | 243  (80.2%) | 1  (0.3%) |
| A3 | PercRemovedManure | 62  (20.5%) | 238  (78.5%) | 3  (1.0%) |
| A4 | PercDisinfectedPersonnel&Vehicle | 63  (20.8%) | 237  (78.2%) | 3  (1.0%) |
| A5 | PercDiseasedChick | 63  (20.8%) | 234  (77.2%) | 6  (2.0%) |
| A6 | PercEnteranceControl | 101  (33.3%) | 185  (61.1%) | 17  (5.6%) |
| A7 | PercAllInAllOut | 83  (27.4%) | 210  (69.3%) | 10  (3.3%) |
| A8 | PercDisinfectedEquipment | 75  (24.7%) | 227  (75.0%) | 1  (0.3%) |
| A9 | PercDisinfectedCage | 58  (19.1%) | 241  (79.6%) | 4  (1.3%) |
| A10 | PercVermitControl | 90  (29.7%) | 204  (67.3%) | 9  (3.0%) |
| A11 | PercChickControl | 91  (30.0%) | 209  (69.0%) | 3  (1%) |
| A12 | PercWater&FeedQuality | 70  (23.1%) | 223  (73.6%) | 10  (3.3%) |
| A13 | PercTransitionZone | 85  (28.0%) | 209  (69.0%) | 9  (3.0%) |
| A14 | PercChickFixedSupply | 91  (30.3%) | 204  (67.3%) | 8  (2.4%) |
| A15 | PercLoadArea | 83  (27.4%) | 213  (70.3%) | 7  (2.3%) |

# **†‘Recognising the importance of biosecurity measures’ included farmers who considered specific biosecurity measures ‘Highly important’ & ‘Important’.**

# **‡‘Not recognising the importance of biosecurity measures’ included farmers who considered specific biosecurity measures ‘Neutral’, ‘Unimportant’ & ‘Highly unimportant’.**

# Table S.4 Descriptive statistics of farmers’ behaviours in relation to biosecurity (n = 303)

| Code | Abbreviation | Desirable action † | Adoption of the desirable action (%) |
| --- | --- | --- | --- |
|
| B1 | DisinfectedChickHouse | Yes | 93.1% |
| B2 | SanitaryPeriod | Yes | 91.4% |
| B3 | VaccineProgramme | Yes | 69.6% |
| B4 | AllInAllOut | Yes | 37.0% |
| B5 | DisinfectedPersonnelEnterance | Yes | 82.5% |
| B6 | DisinfectedVehicle | Yes | 82.8% |
| B7 | BirdNetting | Yes | 36.3% |
| B8 | DisinfectedPersonnel | Yes | 77.2% |
| B9 | VehicleArrivalBroilerEmpty | Yes | 53.1% |
| B10 | DowntimeControl | Yes | 66.7% |
| B11 | TransitionZone | Yes | 58.4% |
| B12 | ChickMovedBetweenHouse | No | 88.4% |
| B13 | LoadArea | Yes | 44.9% |
| B14 | DisinfectedEquipmentBetweenHouse | Yes | 32.0% |
| B15 | DisinfectedEquipmentAfterUse | Yes | 35.0% |
| B16 | DisinfectedEquipmeentBeforeEntering | Yes | 62.7% |
| B17 | DisinfectedNeedleBetweenHouse | Yes | 32.3% |
| B18 | FrequencyChickDisposal | Yes | 63.4% |
| B19 | CageDisinfectedArrivial | Yes | 75.6% |
| B20 | DiseasdChickHandledAfterHealthyChick | Yes | 31.0% |
| B21 | ManureMoved | Yes | 96.7% |
| B22 | CageEntering | Yes | 75.6% |
| B23 | DiseasedChickIsolation | Yes | 35.6% |
| B24 | CageEmpty | Yes | 73.6% |
| B25 | CarcassStored | Yes | 84.2% |
| B26 | ChickFixedSupply | Yes | 54.8% |
| B27 | ManureStored | Yes | 41.9% |
| B28 | WaterQuality | Yes | 5.9% |
| B29 | FeedQuality | Yes | 5.3% |

†The desirable action represented an appropriate biosecurity status.

| Table S.5 Descriptive statistics of the associations of farmers’ biosecurity attitudes and their related biosecurity behaviours  **Group 1: Reject the null hypothesis (**Farmers’ attitudes and behaviours in relation to a specific biosecurity measure were demonstrated to be significantly associated**)**  **A13*B11 (**PercTransitionZone ***** TransitionZone**)**   | Category | Taking the desirable biosecurity action | Not taking the desirable biosecurity action | | --- | --- | --- | | Recognising the importance of biosecurity measures | **146** | **63** | | Not recognising the importance of biosecurity measures | **31** | **50** |   The chi-square statistic is 24.487.  *p*=0.00001   | **A4*B8 (**PercDisinfectedPersonnel&Vehicle *****DisinfectedPersonnel**)** | | |  | | --- | --- | --- | --- | | Category | Taking the desirable biosecurity action | Not taking the desirable biosecurity action | | | Recognising the importance of biosecurity measures | **194** | **43** | | | Not recognising the importance of biosecurity measures | **40** | **23** | |   The chi-square statistic is 9.7815. *p*=0.0018 |
| --- | --- | --- | --- | --- | --- | --- | --- | --- | --- | --- | --- | --- | --- | --- | --- | --- | --- | --- | --- | --- | --- | --- | --- | --- | --- |

**A4*B6 (PercDisinfectedPersonnel&Vehicle * DisinfectedVehicle)**

| Category | Taking the desirable biosecurity action | Not taking the desirable biosecurity action |
| --- | --- | --- |
| Recognising the importance of biosecurity measures | **205** | **32** |
| Not recognising the importance of biosecurity measures | **46** | **16** |

The chi-square statistic is 5.5209.  *p*=0.0188

| **A4*B5 (**PercDisinfectedPersonnel&Vehicle ***** DisinfectedPersonnelEnterance**)** | | |
| --- | --- | --- |
| Category | Taking the desirable biosecurity action | Not taking the desirable biosecurity action |
| Recognising the importance of biosecurity measures | **205** | **30** |
| Not recognising the importance of biosecurity measures | **45** | **18** |

The chi-square statistic is 9.1846.  *p*=0.0024

**A15*B13** (PercLoadArea *****LoadArea**)**

| Category | Taking the desirable biosecurity action | Not taking the desirable biosecurity action |
| --- | --- | --- |
| Recognising the importance of biosecurity measures | **85** | **127** |
| Not recognising the importance of biosecurity measures | **51** | **32** |

The chi-square statistic is 10.9435. *p*=0.0009

| **A10*B7 (**PercVermitControl ***** BirdNetting**)** | | | |
| --- | --- | --- | --- |
| Category | Taking the desirable biosecurity action | Not taking the desirable biosecurity action |  |
| Recognising the importance of biosecurity measures | **74** | **13** |  |
| Not recognising the importance of biosecurity measures | **36** | **22** |  |

The chi-square statistic is 10.0433.  *p*=0.0015

| **A11*B12 (**PercChickControl ***** ChickMovedBetweenHouse**)** | | | |
| --- | --- | --- | --- |
| Category | Taking the desirable biosecurity action | Not taking the desirable biosecurity action |  |
| Recognising the importance of biosecurity measures | **192** | **17** |  |
| Not recognising the importance of biosecurity measures | **76** | **15** |  |

The chi-square statistic is 4.6382. *p*=0.0313

**A8*B16 (**PercDisinfectedEquipment ***** DisinfectedEquipmeentBeforeEntering**)**

| Category | Taking the desirable biosecurity action | Not taking the desirable biosecurity action |
| --- | --- | --- |
| Recognising the importance of biosecurity measures | **158** | **64** |
| Not recognising the importance of biosecurity measures | **32** | **41** |

The chi-square statistic is 17.9065. *p*=0.000023

| **A8*B15** (PercDisinfectedEquipment ***** DisinfectedEquipmentAfterUse**)** | | | |
| --- | --- | --- | --- |
| Category | Taking the desirable biosecurity action | Not taking the desirable biosecurity action |  |
| Recognising the importance of biosecurity measures | **78** | **19** |  |
| Not recognising the importance of biosecurity measures | **28** | **18** |  |

The chi-square statistic is 6.2134. *p*=0.0127

**A8*B14 (**PercDisinfectedEquipment ***** DisinfectedEquipmentBetweenHouse**)**

| Category | Taking the desirable biosecurity action | Not taking the desirable biosecurity action |
| --- | --- | --- |
| Recognising the importance of biosecurity measures | **74** | **22** |
| Not recognising the importance of biosecurity measures | **23** | **23** |

The chi-square statistic is 10.5375. *p*=0.0012

| **A8*B17 (**PercDisinfectedEquipment ***** DisinfectedNeedleBetweenHouse**)** | | | |
| --- | --- | --- | --- |
| Category | Taking the desirable biosecurity action | Not taking the desirable biosecurity action |  |
| Recognising the importance of biosecurity measures | **72** | **26** |  |
| Not recognising the importance of biosecurity measures | **26** | **20** |  |

The chi-square statistic is 4.136. *p*=0.04198

| **A14*B26** (PercChickFixedSupply ***** ChickFixedSupply**)** | | | |
| --- | --- | --- | --- |
| Category | Taking the desirable biosecurity action | Not taking the desirable biosecurity action |  |
| Recognising the importance of biosecurity measures | **123** | **81** |  |
| Not recognising the importance of biosecurity measures | **43** | **47** |  |

The chi-square statistic is 3.9798. *p*=0.0461

| **Group 2: A failure to reject the null hypothesis at the 5% significant level (**Farmers’ attitudes and behaviours in relation to a specific biosecurity measure were not demonstrated to be significantly associated**):**  **A1*B3 (**PercVaccineProgramme ***** VaccineProgramme**)**   | Category | Taking the desirable biosecurity action | Not taking the desirable biosecurity action | | --- | --- | --- | | Recognising the importance of biosecurity measures | **177** | **8** | | Not recognising the importance of biosecurity measures | **34** | **3** |   The Fisher exact test statistic value is 0.2159. *p*=0.333   | The McNemar's test is 14.881.  *p* =0.0001  **A7*B4 (**PercAllInAllOut *****AllInAllOut**)** | | | | | --- | --- | --- | --- | | Category | Taking the desirable biosecurity action | Not taking the desirable biosecurity action |  | | Recognising the importance of biosecurity measures | **78** | **17** |  | | Not recognising the importance of biosecurity measures | **34** | **16** |  |   The chi-square statistic is 3.7076. *p*=0.0542  The McNemar's test is  5.020 .  *p* = 0.0251 | | | |
| --- | --- | --- | --- | --- | --- | --- | --- | --- | --- | --- | --- | --- | --- | --- | --- | --- | --- | --- | --- | --- | --- | --- | --- | --- | --- | --- | --- | --- |
| **A2*B1 (**PercDisinfectedChickHouse ***** DisinfectedChickHouse**)** | | |  |
| Category | Taking the desirable biosecurity action | Not taking the desirable biosecurity action |  |
| Recognising the importance of biosecurity measures | **224** | **17** |  |
| Not recognising the importance of biosecurity measures | **58** | **1** |  |

The Fisher exact test statistic value is 0.2159. *p*=0.2159

| The McNemar's test is 21.333. *p* =0.0001  **A2*B2 (**PercDisinfectedChickHouse ***** SanitaryPeriod**)** | | | |
| --- | --- | --- | --- |
| Category | Taking the desirable biosecurity action | Not taking the desirable biosecurity action |  |
| Recognising the importance of biosecurity measures | **223** | **17** |  |
| Not recognising the importance of biosecurity measures | **54** | **4** |  |

The Fisher exact test statistic value is 1. *p*=1

The McNemar's test is 18.254.  *p* <0.0001

| **A6*B9 (**PercEnteranceControl ***** VehicleArrivalBroilerEmpty**)** | | | |
| --- | --- | --- | --- |
| Category | Taking the desirable biosecurity action | Not taking the desirable biosecurity action |  |
| Recognising the importance of biosecurity measures | **102** | **78** |  |
| Not recognising the importance of biosecurity measures | **59** | **42** |  |

The chi-square statistic is 0.0809. *p*=0.7761

The McNemar's test is 24.174.  *p* <0.0001

| **A6*B10 (**PercEnteranceControl *****DowntimeControl**)** | | | |
| --- | --- | --- | --- |
| Category | Taking the desirable biosecurity action | Not taking the desirable biosecurity action |  |
| Recognising the importance of biosecurity measures | **136** | **59** |  |
| Not recognising the importance of biosecurity measures | **66** | **30** |  |

The chi-square statistic is 0.0299. *p*=0.8627

The McNemar's test is 66.416.  *p* <0.0001

| **A5*B23 (**PercDiseasedChick ***** DiseasedChickIsolation**)** | | | |
| --- | --- | --- | --- |
| Category | Taking the desirable biosecurity action | Not taking the desirable biosecurity action |  |
| Recognising the importance of biosecurity measures | **77** | **20** |  |
| Not recognising the importance of biosecurity measures | **31** | **8** |  |

The chi-square statistic is 0.0002. *p*=0.988996

The McNemar's test is 1.961.  *p*=0.1614

| **A5*B20 (**PercDiseasedChick ***** DiseasdChickHandledAfterHealthyChick**)** | | | |
| --- | --- | --- | --- |
| Category | Taking the desirable biosecurity action | Not taking the desirable biosecurity action |  |
| Recognising the importance of biosecurity measures | **68** | **27** |  |
| Not recognising the importance of biosecurity measures | **26** | **13** |  |

The chi-square statistic is 0.3186*. p*=0.5724

The McNemar's test is 0.000.  *p* =1.0000

| **A12*B29 (**PercWater&FeedQuality *****FeedQuality**)** | | | |
| --- | --- | --- | --- |
| Category | Taking the desirable biosecurity action | Not taking the desirable biosecurity action |  |
| Recognising the importance of biosecurity measures | **10** | **73** |  |
| Not recognising the importance of biosecurity measures | **6** | **52** |  |

The chi-square statistic is 0.0985. *p*=0.7537

The McNemar's test is 55.139. *p* <0.0001

| **A12*B28 (**PercWater&FeedQuality *****WaterQuality**)** | | | |
| --- | --- | --- | --- |
| Category | Taking the desirable biosecurity action | Not taking the desirable biosecurity action |  |
| Recognising the importance of biosecurity measures | **11** | **77** |  |
| Not recognising the importance of biosecurity measures | **7** | **52** |  |

The chi-square statistic is 0.0133*. p*=0.9083

The McNemar's test is  56.679.  *p* < 0.0001

| **A9*B24 (**PercDisinfectedCage ***** CageEmpty ) | | | |
| --- | --- | --- | --- |
| Category | Taking the desirable biosecurity action | Not taking the desirable biosecurity action |  |
| Recognising the importance of biosecurity measures | **163** | **35** |  |
| Not recognising the importance of biosecurity measures | **60** | **14** |  |

The chi-square statistic is 0.0133. *p*=0.8125

The McNemar's test is 6.063.  *p*=0.0138

| **A9*B19 (**PercDisinfectedCage ***** CageDisinfectedArrivial**)** | | |  |
| --- | --- | --- | --- |
| Category | Taking the desirable biosecurity action | Not taking the desirable biosecurity action | |
| Recognising the importance of biosecurity measures | **170** | **36** | |
| Not recognising the importance of biosecurity measures | **59** | **16** | |

The chi-square statistic is 0.5425. *p*=0.4614

The McNemar's test is 5.095.  *p* = 0.0240

| **A9*B22 (**PercDisinfectedCage ***** CageEntering**)** | | |  |
| --- | --- | --- | --- |
| Category | Taking the desirable biosecurity action | Not taking the desirable biosecurity action | |
| Recognising the importance of biosecurity measures | **172** | **33** | |
| Not recognising the importance of biosecurity measures | **57** | **15** | |

The chi-square statistic is 0.8342*. p*=0.3611

The McNemar's test is 5.878.  *p*=0.0153

| **A3*B27** (PercRemovedManure ***** ManureStored**)** | | | |
| --- | --- | --- | --- |
| Category | Taking the desirable biosecurity action | Not taking the desirable biosecurity action |  |
| Recognising the importance of biosecurity measures | **104** | **130** |  |
| Not recognising the importance of biosecurity measures | **23** | **36** |  |

The chi-square statistic is 0.5723*. p*=0.4493

The McNemar's test is   73.438.  *p* <0.0001

**A3*B25 (**PercRemovedManure ***** CarcassStored**)**

| Category | Taking the desirable biosecurity action | Not taking the desirable biosecurity action |
| --- | --- | --- |
| Recognising the importance of biosecurity measures | **207** | **31** |
| Not recognising the importance of biosecurity measures | **48** | **13** |

The chi-square statistic is 2.6565. *p*=0.10313

The McNemar's test is 3.241.  *p* =0.0718

| **A3*B21 (**PercRemovedManure ***** ManureMoved**)** | | | |
| --- | --- | --- | --- |
| Category | Taking the desirable biosecurity action | Not taking the desirable biosecurity action |  |
| Recognising the importance of biosecurity measures | **234** | **2** |  |
| Not recognising the importance of biosecurity measures | **59** | **1** |  |

The chi-square statistic is 0.4945. *p*=0.4945

The McNemar's test is 51.410.  *p* <0.0001

| **A3*B18 (**PercRemovedManure ***** FrequencyChickDisposal**)** | | | |
| --- | --- | --- | --- |
| Category | Taking the desirable biosecurity action | Not taking the desirable biosecurity action |  |
| Recognising the importance of biosecurity measures | **157** | **81** |  |
| Not recognising the importance of biosecurity measures | **35** | **27** |  |

The chi-square statistic is 1.9327. *p*=0.1645

The McNemar's test is 17.457.  p<0.0001

*Note:*

1. *Recognising the importance of biosecurity measures (including the responses of “Highly important & Important” of the five-point Likert scale in terms of the attitude variables)*
2. *Not recognising the importance of biosecurity measures(including the responses of “Neutral& Unimportant &Highly unimportant”of the five-point Likert scale in terms of the attitude variables)*

Reference: Pao, H.N. (2017) *Identifying Human Behavioural and Epidemiological Factors Critical for the Success of a High Biosecurity Compartmentalisation Scheme in Taiwan’s Broiler Industry* (PhD Thesis). Royal Veterinary College, University of London.
